# Identifying involvement of Lys251/Asp252 pair in electron transfer and associated proton transfer at the quinone reduction site of *Rhodobacter capsulatus* cytochrome *bc*_1_

**DOI:** 10.1016/j.bbabio.2016.07.003

**Published:** 2016-10

**Authors:** Patryk Kuleta, Marcin Sarewicz, Pekka Postila, Tomasz Róg, Artur Osyczka

**Affiliations:** a Department of Molecular Biophysics, Faculty of Biochemistry, Biophysics and Biotechnology, Jagiellonian University in Krakow, Gronostajowa 7, 30-387 Kraków, Poland; b Structural Bioinformatics Laboratory, Biochemistry, Faculty of Science and Engineering, Åbo Akademi University, Turku, Finland; c Department of Physics, Tampere University of Technology, P.O. Box 692, FI-33101 Tampere, Finland; d Department of Physics, University of Helsinki, P.O. Box 64, FI-00014 Helsinki, Finland

**Keywords:** Cytochrome *bc*_1_, Mitochondrial complex III, Electron transfer, Proton transfer, Quinone

## Abstract

Describing dynamics of proton transfers in proteins is challenging, but crucial for understanding processes which use them for biological functions. In cytochrome *bc*_1_, one of the key enzymes of respiration or photosynthesis, proton transfers engage in oxidation of quinol (QH_2_) and reduction of quinone (Q) taking place at two distinct catalytic sites. Here we evaluated by site-directed mutagenesis the contribution of Lys251/Asp252 pair (bacterial numbering) in electron transfers and associated with it proton uptake to the quinone reduction site (Q_i_ site). We showed that the absence of protonable group at position 251 or 252 significantly changes the equilibrium levels of electronic reactions including the Q_i_-site mediated oxidation of heme *b*_H_, reverse reduction of heme *b*_H_ by quinol and heme *b*_H_/Q_i_ semiquinone equilibrium. This implicates the role of H-bonding network in binding of quinone/semiquinone and defining thermodynamic properties of Q/SQ/QH_2_ triad. The Lys251/Asp252 proton path is disabled only when both protonable groups are removed. With just one protonable residue from this pair, the entrance of protons to the catalytic site is sustained, albeit at lower rates, indicating that protons can travel through parallel routes, possibly involving water molecules. This shows that proton paths display engineering tolerance for change as long as all the elements available for functional cooperation secure efficient proton delivery to the catalytic site.

## Highlights

• Contribution of D252 and K251 in Q_i_ catalysis of cyt *bc*_1_ is explored by mutagenesis.
• The proton path is disabled only when both protonable groups are eliminated.
• H-bonding network influences thermodynamic properties of Q/SQ/QH_2_ triad in Q_i_ site.
• Parallel routes for proton transfer to the Q_i_ site are implicated.

## 1 Introduction

Proton translocation across energy conserving membrane is crucial for generation of proton motive force. In Peter Mitchell's redox loop mechanism, proton translocation is achieved by a functional coupling of two reactions: an oxidation of quinol with release of two protons at one side of the membrane and a reduction of quinone with uptake of two protons at the opposite side of the membrane [1], [2], [3]. The quinol oxidation and quinone reduction sites can be located in two separate enzymes (bacterial examples [4]), or they can be assembled within one enzyme. The latter case concerns cytochrome *bc*_1_, a key component of many photosynthetic and respiratory systems including mitochondrial respiration [5], [6].

Cytochrome *bc*_1_ is a functional dimer [7]. The quinol oxidation and quinone reduction sites are located within cytochrome *b* subunit, which together with cytochrome *c*_1_ and iron-sulfur (ISP) subunit form the catalytic core of the monomer [8]. The quinol oxidation and quinone reduction sites are named the Q_o_ and Q_i_ sites, respectively. In the Q_o_ site, the oxidation of quinol releases two protons to the intermembrane space. The electrons from this reaction are directed into two separate cofactor chains. The high potential c-chain transfers one electron to cytochrome *c via* iron-sulfur cluster [2Fe-2S], while the low potential b-chain delivers the second electron through hemes *b*_L_ and *b*_H_ to the Q_i_ site. The sequential reduction of quinone to quinol through a semiquinone intermediate (SQ_i_) is associated with an uptake of two protons from the mitochondrial matrix or cytoplasm [9], [10]. It follows that a complete reduction of one quinone molecule at the Q_i_ site requires oxidation of two quinol molecules at the Q_o_ site. In addition, the electron transfer between two hemes *b*_L_ is possible [7], [11], [12], [13]. This secures functional connection of the two Q_o_ and two Q_i_ sites in the dimer.

While the electron paths within cytochrome *bc*_1_ are well defined, the proton paths are much less known. This is in part due to the lack of methods that can directly monitor proton transfers. While uncertainties related with proton transfers concern both the Q_o_ and Q_i_ sites, here we focus just on the Q_i_ site.

Before X-ray structures of cytochrome *bc*_1_ were known, early site-directed mutagenesis successfully identified several key protonable residues associated with the operation of the Q_i_ site [10], [14], [15]. However, the majority of models incorporating the protonation/deprotonation steps at this site were inferred from the inspection of X-ray structures [16], [17], [18]. Complementary studies based on electron paramagnetic resonance spectroscopy provided information on paramagnetic semiquinone bound to the Q_i_ site [19], [20], [21]. In addition, Poisson-Boltzmann electrostatic calculations described redox-linked protonation state changes for this site [22]. All these studies point towards several important polar residues (His217, Asp252, Lys251, Asn221 in bacterial numbering) that can potentially be involved in the substrate binding (Q and SQ_i_) and/or its protonation/deprotonation. Besides these amino acid side chains, cardiolipin (CL) was also postulated to facilitate proton transfers at the entry point from the protein exterior (dimer interface) to the Q_i_ site. In this scenario, CL together with a neighboring lysine residue (Lys251) and water molecules can form the CL/K pathway delivering protons to the site [16], [23], [24].

Our recent MD simulation study [25] suggests that the role of Lys251 is more direct than the prior CL/K pathway hypothesis implied. After acquiring a proton from the dianionic CL head group the positively charged Lys251 could rotate into the Q_i_ site to form a salt bridge with the deprotonated and negatively-charged Asp252 side chain. This fully bent Lys251 conformation, which is not seen in any substrate-bound X-ray crystal structures, results from semiquinone binding in the simulations, but pKa calculations indicate that the switch-like motion would be pH-dependent and possible even without a bound substrate at the Q_i_ site.

The rotation of the Lys251 side-chain implicates the possibility of functional connection between Lys251 and Asp252 for proton transfers to the Q_i_ site. In view of this new finding, we examined the consequences of replacements of Lys251 and Asp252 with non-protonable residues for the functioning of cytochrome *bc*_1_ *in vivo* and for the kinetics of electron and proton transfers. Comparative analysis of separate replacements of either Lys251 or Asp252 side chains (single mutants) and simultaneous replacements of both side chains (double mutants) supports the idea that functional cooperation between Lys251 and Asp252 facilitates proton transfers to the Q_i_ site. It also reveals a limited plasticity of this path to accommodate a lack of one, but not two of protonable groups from the Lys251/Asp252 pair.

## 2 Methods

### 2.1 Mutant preparation

*Rhodobacter* (*R.*) *capsulatus* cells containing substitutions at 251 and 252 positions in cytochrome *b* subunit were obtained using a genetic system originally developed by Dr. F. Daldal [26]. Mutations K251M, D252A, D252N were introduced in the cytochrome *b* gene using QuikChange site-directed mutagenesis system (Stratagene) and the following PCR primers:

D252A_F: 5′-TAT TTC GTG ATC AAG G**CG** CTG TTC GCG CTG GCC-3′;
D252A_R: 5′-CAG CGC GAA CAG **CG**C CTT GAT CAC GAA ATA CGG-3′;
D252N_F: 5′-TTC GTG ATC AAG **A**AC CTG TTC GCG CTG GC-3′;
D252N_R: 5′-AG CGC GAA CAG GT**T** CTT GAT CAC GAA ATA CGG-3′;
K251M_F: 5′-G TAT TTC GTG ATC A**T**G GAC CTG TTC GCG C-3′;
K251M_R: 5′-C GAA CAG GTC C**A**T GAT CAC GAA ATA CGG C-3′;
K251M/D252A_F: 5′-G TAT TTC GTG ATC A**T**G G**CG** CTG TTC GCG CTG GCC C-3′;
K251M/D252A_R: 5′-GC GAA CAG **CG**C C**A**T GAT CAC GAA ATA CGG C-3′;
K251M/D252N_F: 5′-CCG TAT TTC GTG ATC A**T**G **A**AC CTG TTC GCG CTG GCC C-3′;
K251M/D252N_R: 5′-GGC CAG CGC GAA CAG GT**T** C**A**T GAT CAC GAA ATA CGG C-3′.

As a template DNA pPET1 plasmid containing wild type (WT) *petABC* operon was used. The *Bst*XI-*Xma*I fragment of the operon containing the desired mutations, and no other mutations, were inserted into pMTS1 vector and introduced into MT-RBC1 *R. capsulatus* strain using triparental crossing [26]. The presence of introduced mutations was confirmed by sequence analysis of *pet*B gene on plasmid isolated from mutated *R. capsulatus* strains. *R. capsulatus* bacteria were grown under semiaerobic or photoheterotrophic conditions as described previously [27]. To test for the occurrence of reversion mutations, 100 μl of 1 l overnight liquid culture of the mutant strains were spread on mineral-peptone-yeast extract (MPYE) plates and kept in selective photosynthetic cultures for 12 days. Single colonies that acquired the Ps^+^ phenotype (photosynthetic competence) were isolated, and reversion mutations were identified by sequencing the entire *pet*ABC operon.

### 2.2 Isolation of chromatophores and protein purification

Procedure described previously in ref. [28] was used to obtain the chromatophore membranes from *R. capsulatus* cells growing under semiaerobic conditions. After isolation, chromatophores were homogenized and suspended in MOPS pH 7.0 or Tris pH 9.0 buffer (for light-induced electron transfer measurements) or in 50 mM Tris pH 8.0 buffer containing 100 mM NaCl, 0.01% DDM and 20% glycerol (for protein purification). Cytochrome *bc*_1_ complexes were isolated from detergent-solubilized chromatophores using ion-exchange chromatography (DEAE-BioGel A) as described [28].

### 2.3 Light-induced electron transfer measurements

Double-wavelength time-resolved optical spectrophotometer [29] was used to measure the kinetics of electron transfer through hemes of cytochrome *bc*_1_ in chromatophores. Transient kinetics of hemes *b* were measured at 560–570 nm after activation by single saturating flash (~ 10 μs). Measurements were performed at pH 7.0 (50 mM MOPS, 100 mM KCl, 1 mM EDTA) or pH 9.0 (50 mM Tris, 100 mM KCl, 1 mM EDTA) under conditions of low (100 mV) or high (200 mV, 250 mV) ambient redox potential. Experiments were performed under anaerobic conditions in the presence of redox mediators and valinomycin as described in [29] except the carotenoid bandshift measurements for which the valinomycin was omitted. The rates of flash-induced electron transfer reactions were calculated from single exponential function fitted to: heme *b*_H_ reduction in the presence of antimycin, *b*_H_ re-oxidation without inhibitors and to heme *b*_H_ reduction from reverse reaction in the presence of myxothiazol (Table 1).

### 2.4 EPR measurements of semiquinone

CW EPR spectra of semiquinone were obtained for isolated cytochrome *bc*_1_ complexes. Samples of WT and mutants were measured at 200 K in 50 mM Tris buffer pH 8.0 containing 100 mM KCl, 0.01% DDM and 1 mM EDTA. All spectra were obtained using the following parameters: microwave frequency – 9.39 GHz, sweep width - 180 G, modulation amplitude – 10 G, microwave power – 1.9 mW. Semiquinone was generated in samples by incubation of 50 μM cytochrome *bc*_1_ with myxothiazol (Q_o_ site inhibitor) and subsequent addition of 2,3-dimethoxy-5-methyl-6-decyl-1,4-benzohydroquinone (DBH_2_) as a substrate. The negative control was obtained by addition of antimycin (Q_i_ site inhibitor) to samples treated previously with myxothiazol and DBH_2_. Both DBH_2_ and myxothiazol were used at final concentration of 200 μM while antimycin was used at 400 μM. Quantitative EPR analysis of the semiquinone was performed using 4-Hydroxy-TEMPO (TEMPOL) as a standard as described in [30]. To obtain the calibration curve, TEMPOL was measured under the same buffer, temperature and EPR parameters conditions as those used for SQ_i_ measurements.

## 3 Results

### 3.1 General biochemical and phenotypic properties of mutants of D252 and K251

**Fig. 1:**
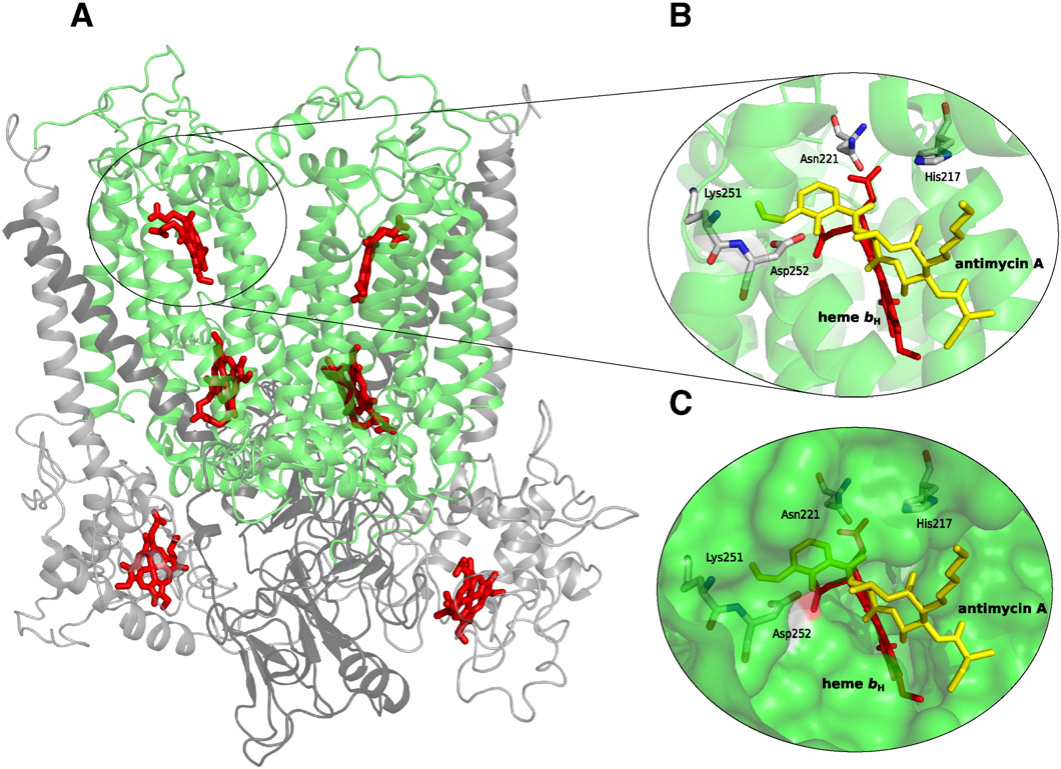
A - crystal structure of dimeric cytochrome *bc*_1_ from *R. capsulatus* (PDB: 1ZRT) [8]. Subunits in both monomers are: cytochrome *b* - green, cytochrome *c*_1_ - light gray, ISP subunit - dark gray. Hemes are red sticks. B – close-up view of the Q_i_ site with antimycin (yellow sticks) bound at the site. The protonable residues in the vicinity of the quinone binding site are indicated. C - view of the Q_i_ site as in B with molecular surface added to visualize the entrance to the cavity. B and C show the structure of the Q_i_ site with antimycin from *R.sphaeroides* (PDB: 2QJP) [50].

**Fig. 2:**
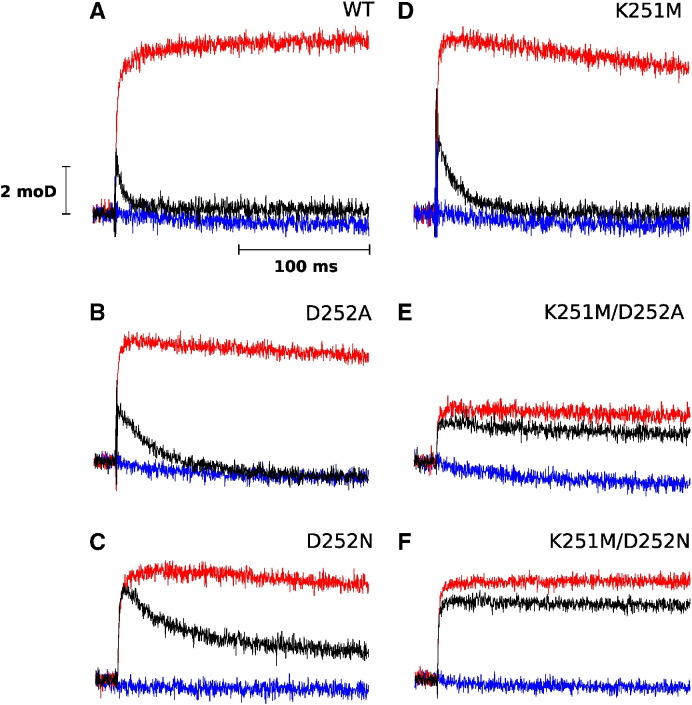
Light-induced heme *b* reduction and re-oxidation under low ambient redox potential. Transient kinetics at 560–570 nm were followed for WT (A), single mutants D252A (B), D252N (C), K251M (D) and double mutants K251 M/D252A (E), K251M/D252N (F). Traces were recorded without inhibitors (black), after inhibition with antimycin (red), and subsequent inhibition with myxothiazol (blue) at pH 7 and ambient potential of 100 mV.

**Fig. 3:**
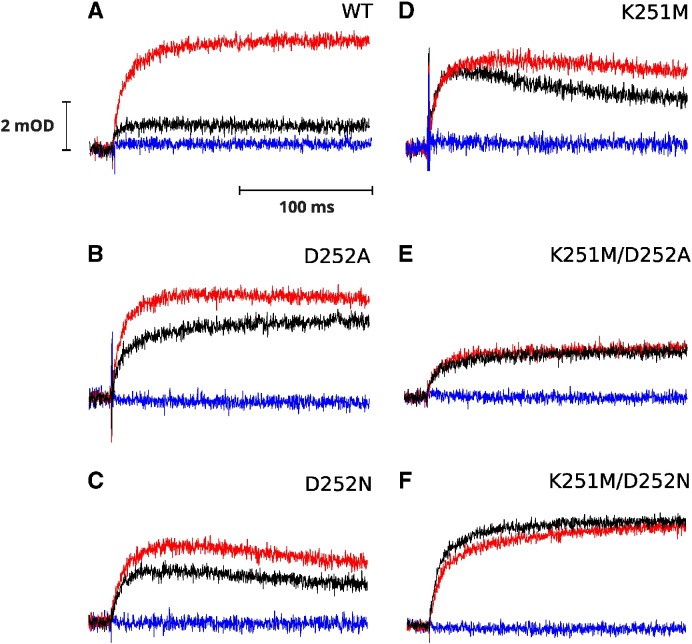
Light-induced heme *b* reduction under high ambient redox potential. Transient kinetics at 560–570 nm were followed for WT (A), single mutants D252A (B), D252N (C), K251M (D) and double mutants K251M/D252A (E), K251M/D252N (F). Traces were recorded without inhibitors (black), after inhibition with antimycin (red), and subsequent inhibition with myxothiazol (blue) at pH 7 and ambient potential of 200 mV.

**Fig. 4:**
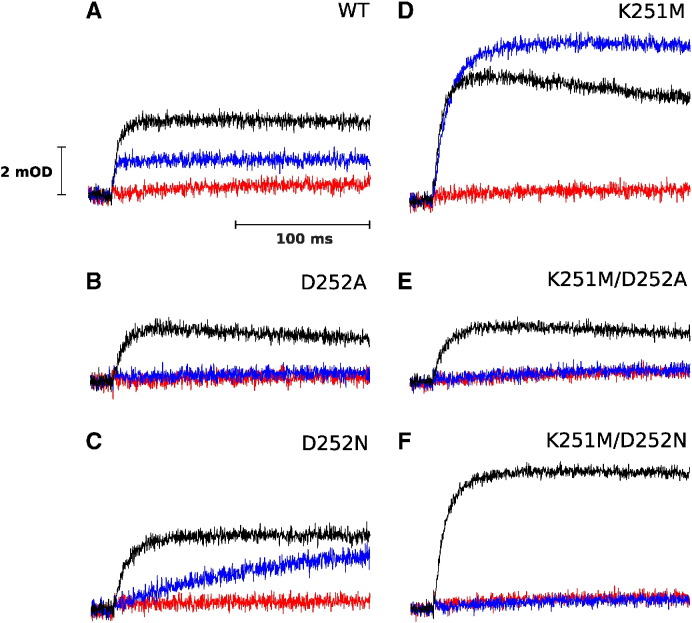
Reduction of heme *b*_H_ through reverse electron transfer at the Q_i_ site. Transient kinetics at 560–570 nm were followed for WT (A), single mutants D252A (B), D252N (C), K251M (D) and double mutants K251M/D252A (E), K251M/D252N (F). Traces were recorded without inhibitors (black), with myxothiazol (blue), and with both myxothiazol and antimycin present (red) at pH 9 and ambient potential of 250 mV.

**Fig. 5:**
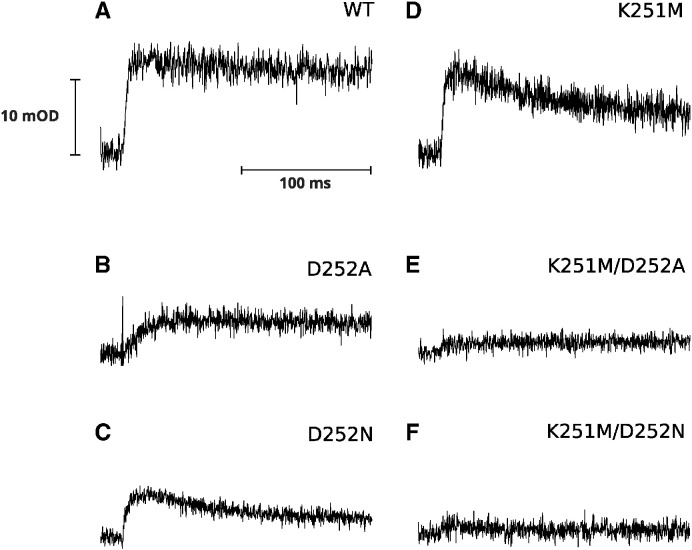
Antimycin-sensitive component of carotenoid bandshift measured for WT (A), D252A (B), D252N (C), K251M (D), K251M/D252A (E), K251M/D252N (F) at pH 7 and ambient potential of 100 mV. Traces were obtained by subtracting transients at 475–490 nm measured in the presence of antimycin from the transients measured without inhibitors.

**Fig. 6:**
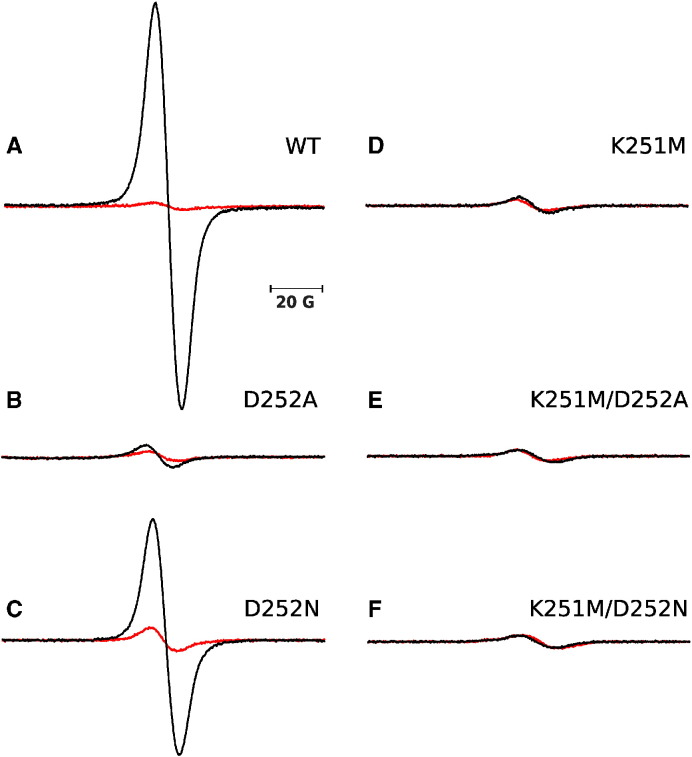
CW EPR spectra of semiquinone radical in isolated cytochrome *bc*_1_. Spectra measured for WT (A), D252A (B), D252N (C), K251M (D), K251M/D252A (E), K251M/D252N (F) at pH 8 with excess of DBH_2_ in the presence of myxothiazol (black). Spectra of the same samples subsequently inhibited with antimycin are shown in red. Conditions of measurements are in the Methods section.

**Fig. 7:**
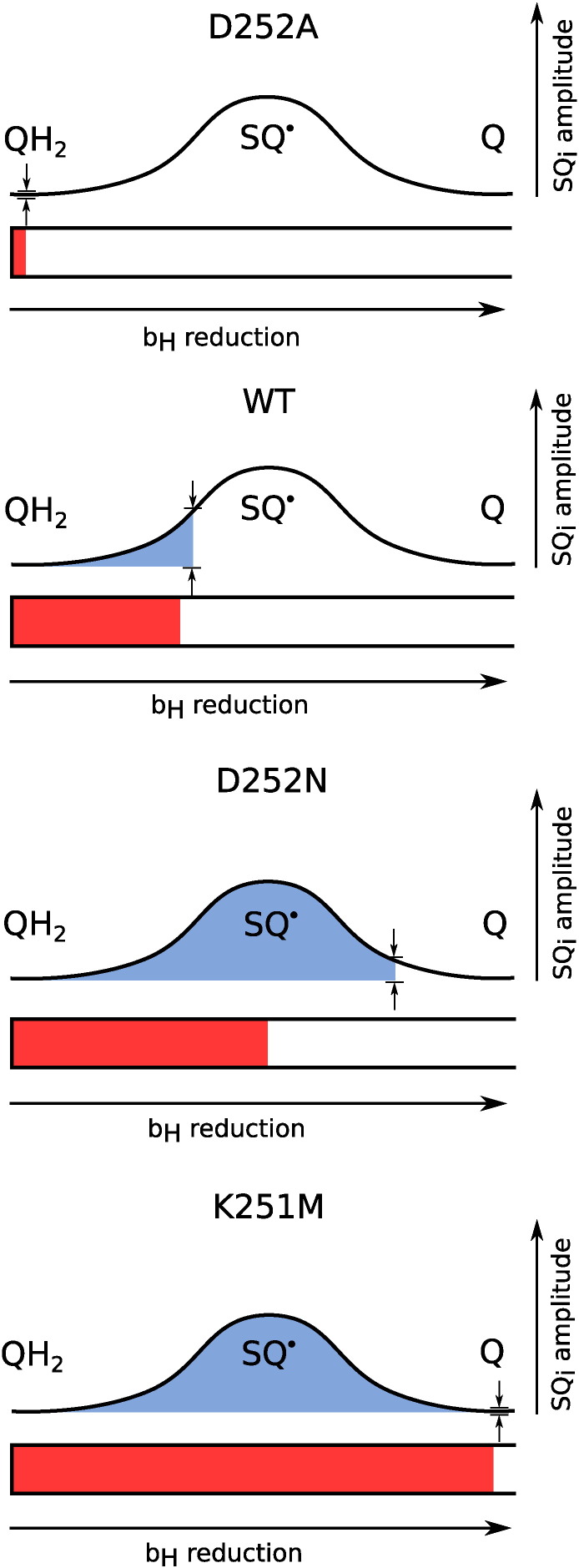
Electron distribution between QH_2_/SQ/Q and heme *b*_H_ in WT and mutants. The length of the red bars (X-axis) indicate level of heme *b*_H_ reduction. Blue areas represent the oxidation level of QH_2_ (more blue as more quinones occupy the Q_i_ site). The relative level of SQ_i_ is showed as amplitude between small arrows (Y-axis).

Conclusions drawn from MD simulations described by Postila et al. [25] and other studies [10], [18], [19] point out four important side chains in SQ binding: Lys251, Asp252, Asn221 and His217 (Fig. 1B). From those we chose Lys251 and Asp252 for experimental testing through site-directed mutagenesis. For this purpose we constructed three single mutants K251M, D252A, D252N and two double mutants K251M/D252A, K251M/D252N. The rationale behind the substitutions of Lys to Met and Asp to Asn was to change the protonable side chains into the non-protonable ones with minimal structural distortions. The substitution of Asp to Ala also tested the removal of protonable group with, possibly, additional structural effects. The properties of those mutants and the most insightful kinetic data are summarized in Table 1 and Fig. 2, Fig. 3, Fig. 4, Fig. 5, Fig. 6, Fig. 7.

The electrophoretic analysis of isolated complexes indicated that in all cases the mutant cells expressed cytochrome *bc*_1_ with all three catalytic subunits (SDS-page profiles showed the presence of three bands corresponding to cyt *c*_1_, cyt *b* and the FeS subunit). The difference optical spectra of all mutated complexes in the isolated form were similar to that of the native complex. The ability to grow under photosynthetic (Ps) conditions, which tests functionality of cytochrome *bc*_1_ *in vivo* [12], [13], [26], [31], [32] indicated that among the mutants only K251M showed a Ps + growth rate comparable to WT (Table 1). D252A showed a very weak Ps growth indicating severe functional impediment. The Ps growth in D252N was better than D252A, however still less robust than that of WT. Both double mutants did not grow under photosynthetic conditions indicating that cytochrome *bc*_1_ is not functional *in vivo* (Table 1).

Incubation of D252A under photosynthetic conditions allowed us to isolate single colonies that exhibited faster Ps growth than original D252A. The DNA sequence analysis of these cells revealed that Ala at position 252 was replaced by Glu. In addition, the reversions were observed for the double mutants: K251M/D252A or K251M/D252N regained Ps + phenotype by placing Glu or Asp at position 252, respectively (Table 1).

### 3.2 Kinetics of light-induced electron transfer

To assay the Q_i_ site function in the mutants we analyzed the rates and amplitudes of light-induced electron transfer in chromatophore membranes under various redox conditions in the absence or presence of inhibitors specifically inactivating Q_o_ or Q_i_ sites [29], [33], [34]. Kinetic transients shown in Fig. 2 compare redox changes of heme *b*_H_ (measured at 560–570 nm) under ambient redox potential setting hemes *b* oxidized and the quinone pool half-reduced prior to flash activation. Under these conditions, heme *b*_H_ in the native enzyme undergoes light-induced reduction followed by re-oxidation (Fig. 2A, black trace). The reduction phase is associated with the oxidation of quinol at the Q_o_ site. The re-oxidation phase occurs through the action of the Q_i_ site (reduction of quinone to semiquinone and then semiquinone to quinol) and is blocked by antimycin, a potent inhibitor of this site (Fig. 2A, red trace) [35]. In the presence of both antimycin and myxothiazol (inhibitor of the Q_o_ site [36]) the enzyme is fully blocked and changes in the redox state of heme *b*_H_ do not occur (Fig. 2A, blue trace). The kinetic transients shown in Fig. 2 indicate that the mutants do not impede the reduction phase observed in the presence of antimycin (red traces in Fig. 2, and rates in Table 1). However, the re-oxidation phase observed in the absence of any inhibitor is clearly slowed down or blocked (Fig. 2, black traces, and rates in Table 1). In the group of single mutants D252A and D252N showed approximately six fold decrease in the rate of this phase, comparing to WT while in K251M, the slowing was less severe (did not exceed two times). In double mutants (K251M/D252A, K251M/D252N), re-oxidation of hemes *b* did not occur on a millisecond timescale (Table 1).

Kinetic transients shown in Fig. 3 compare redox changes of heme *b*_H_ under ambient redox potential setting hemes *b* and quinone pool oxidized prior to flash activation. Under these conditions the amount of quinol molecules after flash activation is limited and approximately only one quinol is oxidized in every Q_o_ site. This leads to reduction of heme *b*_H_ which equilibrates with the occupant of the Q_i_ site. This equilibration is reflected in a difference in amplitudes of heme *b*_H_ reduction in the absence and presence of antimycin (black and red, respectively). While the reduction rates in the presence of antimycin in all mutants are similar and comparable to WT (Table 1) the level of heme *b*_H_ reduction in the absence of any inhibitors is elevated in the mutants. In single mutants (K251M, D252A, D252N) this level approaches approximately 70% of the maximum reduction level (seen in the presence of antimycin), in the double mutants, it reaches the maximum reduction level (the amplitude of black and red trace are comparable).

Kinetic transients shown in Fig. 4 (blue traces) monitor the electron transfer from QH_2_ to heme *b*_H_ at the Q_i_ site (reverse reaction) under conditions where the Q_o_ site is blocked by myxothiazol and the reduction power of Q pool is increased (by increasing pH). Reduction of heme *b*_H_ under these conditions is not observed on a millisecond time scale in D252A and in both double mutants. In D252N this reaction is 70 times slower than in WT (see the rates in Table 1). In K251M, the slowing of the rate is not as severe as in D252N (5 times). At the same time, the amplitude of reverse heme *b*_H_ reduction in K252M is much higher and, unlike in WT, exceeds the amplitude of heme *b* reduction in the absence of inhibitors (compare blue *vs* black in WT and K251M).

### 3.3 Monitoring electrogenic reactions associated with cytochrome *bc*_1_

To get information on proton uptake from bulk solution to the Q_i_ site, we conducted a series of measurements of electrogenic reactions associated with the operation of cytochrome *bc*_1_ by following the antimycin-sensitive phase of carotenoid bandshift (Fig. 5 and Table 1) [37], [38]. In K251M this phase is comparable to WT. D252A and D252N show decrease in the amplitude of this phase which in D252A additionally has a clearly slower rate. In contrast to single mutants, both double mutants (K251M/D252A, K251M/D252N) do not reveal antimycin-sensitive phase of carotenoid bandshift.

### 3.4 Testing the SQ_i_ levels by EPR

Semiquinone in the Q_i_ is observed by EPR as antimycin-sensitive radical signal with g_x_ transition – 2.004 (Fig. 6). Typically, the signal is generated in the samples of isolated cytochrome *bc*_1_ exposed to excess of quinol in the presence of myxothiazol. These conditions favor reverse reaction in the Q_i_ site in which reduction of heme *b*_H_ by QH_2_ leads to formation of stable SQ_i_ [9], [19], [39], [40], [41]. Fig. 6 shows that under these conditions (and with comparable concentrations of cytochrome *bc*_1_) clear SQ_i_ signal can be observed only in WT and D252N (Fig. 6A, C). Quantitative estimation of SQ_i_ concentration indicated the ratio [SQ_i_]/[cytochrome *bc*_1_] of 0.34 and 0.18 for WT and D252N, respectively. Traces of SQ_i_ signals were observed in D252A while no SQ_i_ signal was detected in K251M and double mutants (K251M/D252A, K251M/D252N).

## 4 Discussion

### 4.1 Experimental evidence for involvement of Lys251 and Asp252 in electron/proton reactions in the Q_i_ site

The roles of Lys251 and Asp252 in proton management of the Qi site, suggested by MD simulations [25] are supported by the effects of mutations observed here and in previous studies [16], [18], [19]. The results consistently indicate that mutating Lys251 and/or Asp252 alters the operation of the Q_i_ site without much influence on the Q_o_ site.

The unaffected Q_o_ site was inferred from little influence of the mutations on the rates of Q_o_ site-mediated heme *b*_H_ reduction (Fig. 2, Fig. 3, red traces). The influence of mutations on the Q_i_ site was revealed by various changes in both the electron transfer reactions associated with redox reactions of the Q_i_ site and cytochrome *bc*_1_-related proton translocation. The observation that the rate of the re-oxidation of heme *b*_H_ (Fig. 2, black traces) was slowed down (single mutants) or blocked (double mutants) indicates impediments in electron and proton reactions that involve first electron transfer from heme *b*_H_ to Q and subsequent electron transfer from heme *b*_H_ to SQ to complete Q reduction.

Similar slowing of the re-oxidation of heme *b*_H_ was observed in K251M mutant of *R. sphaeroides*, but not in the other mutant at this position (K251I) for which the kinetics comparable to WT were reported [14]. The two mutants of Asp252 (D252A and D252N) in this species exhibited lack of heme *b*_H_ re-oxidation in the light-induced kinetics in the absence of inhibitors [14]. This was clearly a more severe impediment comparing to the respective mutants shown here.

The redox equilibrium level between heme *b*_H_ and Q or SQ was shifted in the mutants towards reduction of heme *b*_H_ in comparison to WT (Fig. 3, black *vs* red traces), implicating that heme *b*_H_ in mutants faces difficulty in delivering electron to quinone occupying the Q_i_ site. This effect is apparently not a result of a changing in the redox midpoint potential (E_m_) of heme *b*_H_ given the values of E_m_ determined by redox potentiometry (Table 1). These changes of equilibrium are also evident from the measurements of reverse reactions at the Q_i_ site, associated with electron transfer from quinol to oxidized heme *b*_H_ (Fig. 4).

For all these mutants the process of proton uptake from bulk solutions to the Q_i_ site in the mutants, was inferred from the measurements of blue-shift of absorption spectra of carotenoids (carotenoid bandshift) upon generation of transmembrane electric field. The antimycin-sensitive phase of carotenoid bandshift is associated with the action of cytochrome *bc*_1_ complex. Concerning the previous studies [38], [42], [43], [44] and our results we assume that this phase reflects the reactions associated with two protons uptake from aqueous phase into the Q_i_ site after the full quinone reduction is completed. This concerns protonation of oxygen atoms at both the C-1 (through the K251/D252 path) and C-4 groups (through the H217 path) of reduced quinone.

In light of this assumption, the diminished amplitude of the carotenoid bandshift phase in D252A and D252N, and additional slowing in D252A, reflect overall difficulty in uptake of protons to the Q_i_ site, while the elimination of this phase in double mutants indicate much more severe blocking of this process. Single K251M does not influence much the proton uptake, as indicated by similar rate and amplitude of the carotenoid bandshift phase in this mutant (comparing to WT). The mutants of Asp252 in *R. sphaeroides* also affected this phase: D252N showed a slowing, with diminished amplitude while in D252A this phase was abolished. K251M showed a slower phase without amplitude change. In all three cases, changes in the carotenoid bandshift appear to be more severe in *R. sphaeroides* than the effects of respective mutants shown here [14]. They, however, seem to reflect the same phenomenon: perturbed proton transfers to the Q_i_ site.

This, in view of electron transfer measurements, MD simulations and crystal structure data, is most likely associated with the hampered K251/D252 path affecting protonation of quinone C-1 carbonyl. The role of His217 in C-4 carbonyl protonation is inferred from previous studies which showed that replacing His217 to Asp or Arg yielded enzymatically active complexes functional *in vivo* but replacement to Leu deactivated the enzyme leading to loss of its functional competence *in vivo* [10]. Interestingly, H217L fully abolished the antimycin-sensitive phase of carotenoid bandshift, similarly to the effects of double mutants reported here. Thus, the lack of this phase in H217L or double mutants suggests that blocking of just one proton path (either K251/D252 path or H217 path) eliminates the proton uptake in both paths, implicating functional coupling (connection) between them.

We note that, if this and other mutational works including [9], [14], are considered, there is a correlation between the occurrence of antimycin-sensitive carotenoid bandshift phase and the functionality of cytochrome *bc*_1_ *in vivo*: only mutants that show this phase at measurable rates and amplitudes are able to grow photosynthetically. This is understandable, if one considers that the efficiency of proton transfers ultimately defines proton motive generating capacity of the enzyme *in vivo*. This further substantiates the notion that this phase reflects the protons uptake from aqueous phase into the Q_i_ site.

Additional indication for involvement of D252 in proton transfer came from the observation that barely functional D252A and non-functional K251M/D252A or K251M/D252N mutants regained functionality by restoring protonable group (either E or D) at position 252 (Table 1).

### 4.2 The role of H-bonding network in binding of quinone/semiquinone and defining thermodynamic properties of Q/SQ/QH_2_ triad

Considering all kinetic traces shown in (Fig. 2, Fig. 3, Fig. 4), the data from measurements of carotenoid bandshift (Fig. 5) and the EPR data on SQ_i_ (Fig. 6) we may draw the general conclusions on the influence of the mutations on changing the equilibrium of electron transfer and associated with it protonation/deprotonation within the Q_i_ site. The most obvious results are found for the double mutants for which the mechanistic picture is rather simple. Removing of two important protonable side chains within the Q_i_ site exerts a synergistic effect on both electron transfer (there is neither Q/SQ reduction in forward mode (Fig. 2E, F) nor QH_2_ oxidation *via* reverse reaction (Fig. 4E, F) nor detectable SQ_i_ (Fig. 6E, F)) and proton transfer (no observable cytochrome *bc*_1_-mediated proton transfers from outside of the protein to the Q_i_ site (Fig. 5E, F)). All these effects could result from a lack or improper binding of substrate at the site.

The more complex effects are associated with single replacements of either K251 or D252 with non-protonable amino acids. Although the reactions associated with electron transfer between Q or QH_2_ and heme *b*_H_ are generally similar for K251M, D252A and D252N we notice some differences that result from different effect of Lys and Asp on Q/SQ/QH_2_ binding and proton transfer between protein interior and exterior. The sharpest differences between Lys and Asp mutants become visible when analyzing traces in which only theoretically one-electron reactions are involved. It is clear that when Q is awaiting electron from heme *b*_H_ in all three mutants K251M, D252A or D252N the electron is mostly retained at the level of heme *b*_H_ as if the potential of Q/SQ couple was lowered. For K251M, it may reflect a higher degree of deprotonation of Asp carboxyl group that cannot be stabilized by interaction with amine group of Lys which leads to destabilization (weaker binding) of Q or SQ within the Q_i_ site. This destabilization seems to be even more severe for mutant having Asp replaced with non-protonable residues (D252A and D252N) for which there is no direct partner for quinone or semiquinone that may deliver proton and stabilize the binding.

Interestingly, when considering reverse reaction (QH_2_ oxidation by heme *b*_H_ in the Q_i_ site) the differences between the mutants shed light on the proton reactions associated with the SQ/QH_2_ couple. A lack of QH_2_ oxidation in D252A mutant indicates that deprotonation of QH_2_ is blocked when direct proton exchanger (Asp) is replaced by hydrophobic residue. As a result, the semiquinone at the Q_i_ site cannot be effectively formed (Fig. 6B) nor detectable heme *b*_H_ reduction is observed (Fig. 4B). This is even though the proton path from the site to the bulk still exists (with the help of Lys251). D252N mutant encounters similar difficulty, yet the reverse reaction follows but at a very slow rate when compared to WT. In contrast to Ala in D252A, the polar Asn does not repel water molecules from the vicinity of quinone. They, in turn, may alleviate the lack of COO^-^ group of Asp, however they are not as efficient in proton exchange as the K251/D252 pair. Thus, the reverse reaction leads to the reduction of heme *b*_H_. This reaction is two orders of magnitude slower than WT but proceeds to higher level (Fig. 4C, Table 1). Correspondingly, clear EPR signal of SQ_i_ can be detected in this mutant, although its amplitude is lower, when compared to WT (Fig. 6C). In K251M, unlike in D252A or D252N, the efficiency of reverse reaction is unexpectedly high, exceeding the level of WT, as if the interior of the protein was much more alkaline. To explain this, we assume that amine group of Lys251 in WT stabilizes “proper” protonation of Asp carboxyl group and the removal of the amine group in the mutants promotes fast deprotonation of SQ/QH_2_ within the site. Consequently, protons from QH_2_ are sequentially removed with a help of Asp and then full deprotonation promotes transfer of two electrons to the b-chain yielding high level of reduced hemes *b*. This apparent lowering of the redox potential of QH_2_/SQ/Q triad, induced by a very efficient deprotonation, leads to disappearance of the semiquinone EPR signal (Fig. 6D) due to the fact, that upon reverse reaction, the Q_i_ site is overwhelmingly occupied by Q instead of being occupied by QH_2_ or SQ.

In summary, the changes in electron transfer drawn from the reverse reactions associated with different deprotonation reactions allow us to make a general picture of possible equilibration states of Q_i_-site occupant and heme *b*_H_ (Fig. 7). Single mutant D252A and double mutants K251M/D252A and K251M/D252N show neither semiquinone signal nor reduced heme *b*_H_ as the impaired deprotonation of QH_2_ prevents any efficient reactions in the site. In WT, Asp252 side chain interacting with K251 allows the deprotonation of QH_2_ promoting a generation of relatively high level of SQ and moderate level of heme *b*_H_ reduction. It can be envisaged that in this case amount of QH_2_ oxidized to SQ equals the amount of reduced heme *b*_H_. In D252N the deprotonation is even more efficient than in WT, however this is not associated with an elevated level of SQ. This is simply because the electronic equilibrium is shifted from SQ to heme *b*_H_ yielding lower amplitude of SQ and higher level of *b*_H_ heme reduced. In this case more than one electron from QH_2_ is transferred to the b-chain. In K251M, two protons are removed from the vicinity of the bound QH_2_ of SQ which leads to the most efficient reverse reaction - two electrons from QH_2_ eventually go to the b-chain. Thus in equilibrium the Q_i_ site is occupied by Q instead of SQ while the level of reduced heme *b*_H_ is the highest among the tested cytochrome *bc*_1_ forms.

### 4.3 Parallel routes for proton transfer to the Q_i_ site

In several studies, Lys251 and Asp252 have been considered as good candidates for residues securing proton delivery from the peripheral CL to the C-1 carbonyl of quinone [16], [18], [19], [20], [22], [24]. The possible cooperation of these two residues in proton transfer became most evident in recent MD simulations which demonstrated that the side chain of Lys251 can rotate from the periphery of the complex towards the Q_i_ site where formation of a salt bridge with the side chain of Asp252 is possible. In view of this observation, the most obvious scenario leading to protonation of the C-1 carbonyl of quinone involves a sequential protonation of Lys251 and Asp252, as described in detail by Postila et al. [25].

We emphasize, however, that in light of experimental results, any scenario assuming a sequential mechanism of transfer of protons involving Lys251 and Asp252 should be considered as a possible, but certainly not the unique path available for protons to enter the Q_i_ site. Alternative pathway/pathways omitting either Lys251 or Asp252 must exist in single mutants having non-protonable side chains at either of these positions (K251M or D252N), as these mutants still retain much of the electron and proton transfer capabilities and remain functional *in vivo*. This could be result of another protonable group/groups, possibly water molecules, taking over the function of the original side chains that are missing in the mutants, or a reminiscence of natural existence of parallel (multiple) paths for protons in native protein [45]. The latter explanation is quite reasonable in light of the multiplicity for proton paths considered in the case of other quinone binding sites, such as the Q_B_ site of photosynthetic reaction center [46], [47], [48]. However, the double mutants show that the simultaneous presence of non-protonable side chains at both positions (K251M/D252A, K251M/D252N) effectively deactivates proton entry to the Q_i_ site which yields mutants non-functional *in vivo* with fully inactive Q_i_ site. This indicates that at least one of the protonable side chains at either position 251 or 252 must by present. In addition, in *R. sphaeroides* it was observed that the inversion of charges at positions 251 and 252 (double mutant K251D/D252K) had little effect on enzymatic activity and did not affect the function of enzyme *in vivo* [49]. This all indicates that proton paths in this system display engineering tolerance for change as long as all the elements available for functional cooperation secure efficient proton delivery to the catalytic site.

## Transparency document

Transparency document. Image 2

## Figures and Tables

**Table 1:** Selected properties of cytochrome *bc*_1_ mutants.

	Phenotype^a^	Reversions	Light-induced heme *b* reduction	Light-induced heme *b* re-oxidation	Heme *b* reduction from Q_i_ reverse reaction	Carotenoid bandshift phase	E_m_ of hemes	
*b*_H_	*b*_L_
s^− 1^	s^− 1^	s^− 1^	[mV]	
WT	+++	−	1040	185	400	+	35	− 120
D252A	− (+)	D252E	1120	32	0	+	43	− 118
D252N	++	−	830	25	6	+	28	− 107
K251M	+++	−	1080	100	80	+	36	− 109
K251M/D252A	−	K251M/D252E	1250	0	0	−	nd^b^	nd
K251M/D252N	−	K251M	1200	0	0	−	nd	nd

a +++, indicates Ps growth comparable to WT; ++, indicates Ps growth slower than WT (colonies appear on Ps plates with approximately one day delay comparing to WT); − (+), indicates very weak Ps growth (small colonies appear with approximately five days of delay comparing to WT).

b nd, not determined.
